# Involvement of the Avian Dorsal Thalamic Nuclei in Homing Pigeon Navigation

**DOI:** 10.3389/fnbeh.2017.00213

**Published:** 2017-11-02

**Authors:** Paulo E. Jorge, Belmiro V. Pinto, Verner P. Bingman, John B. Phillips

**Affiliations:** ^1^MARE - Marine and Environmental Sciences Centre, ISPA - Instituto Universitário, Lisbon, Portugal; ^2^SIM - Laboratory for Systems Instrumentation and Modeling in Science and Technology for Space and the Environment, Faculdade de Ciências da Universidade de Lisboa, Lisbon, Portugal; ^3^Department of Psychology and J.P. Scott Center for Neuroscience, Mind and Behavior, Bowling Green State University, Bowling Green, KY, United States; ^4^Department of Biological Sciences, Virginia Tech, Blacksburg, VA, United States

**Keywords:** anterior thalamic nuclei, path-based information, olfactory activation, vertebrates, avian navigation, Homing pigeons

## Abstract

The navigational ability of birds has been a focus of popular and scientific interest for centuries, but relatively little is known about the neuronal networks that support avian navigation. In the brain, regions like the piriform cortex, olfactory bulbs, hippocampal formation, vestibular nuclei, and the wulst, are among the brain regions often discussed as involved in avian navigation. However, despite large literature showing a prominent role of some anterior and dorsal thalamic nuclei in mammalian spatial navigation, little is known about the role of the thalamus in avian navigation. Here, we analyzed a possible role of the dorsal anterior thalamic nuclei in avian navigation by combining olfactory manipulations during the transport of young homing pigeons to a release site and c-Fos immunohistochemistry for the mapping brain activity. The results reveal that odor modulated neurons in the avian dorsolateral lateral (DLL) subdivision of the anterior thalamic nuclei are actively involved in processing outward journey, navigational information. Outward journey information is used by pigeons to correctly determine the homeward direction. DLL participation in acquiring path-based information, and its modulation by olfactory exposure, broadens our understanding of the neural pathways underlying avian navigation.

## Introduction

Spatial orientation underlies all aspects of animal behavior. When exploring unfamiliar surroundings, many animals gather information about the path that they follow in order to keep track of their spatial position (Mittelstaedt and Mittelstaedt, 1980; Wiltschko and Wiltschko, 1982; Müller and Wehner, 1988; Bingman and Cheng, 2005; McNaughton et al., 2006). In a wide variety of animals atmospheric odors have been hypothesized to provide a major source of spatial information for long distance navigation (reviewed in Bingman and Cheng, 2005; Wallraff, 2005; Alerstam, 2006), but the role of olfactory cues in avian navigation remains controversial (Wiltschko, 1996; Freake et al., 2006; Jorge, 2011; Phillips and Jorge, 2014).

Behavioral and neurophysiological studies have proposed two fundamentally distinct, but not mutually exclusive roles for odors: (1) “Navigational map hypothesis” discrete sources of natural odors (e.g., olfactory landmarks) that animals encounter while traveling, or being displaced, into unfamiliar surroundings, can be used to help keep track of their path of movement (e.g., Papi and Casini, 1990; Patzke et al., 2010; reviewed in Wallraff, 2005); and (2) “Activation hypothesis” unfamiliar odors could function as an activator of neuronal circuits that process non-olfactory spatial information, i.e., non-home odors could activate neuronal circuits that respond to and integrate navigational information from multiple sensory modalities (e.g., Jorge et al., 2009, 2010, 2014, 2016).

Odors have been shown to play an important role in homing pigeon navigation (either activational and/or navigational; e.g., Phillips and Jorge, 2014; Wallraff, 2014). Access to odors is important during the outward journey, at an unfamiliar site prior to release, and on the flight home (e.g., Jorge et al., 2009, 2010). Additionally, one complicating aspect associated with the processing of olfactory information for navigation is that the processing has a strong lateralized property (e.g., Gagliardo et al., 2005, 2007; Patzke et al., 2010). In birds that are homing, olfactory input triggers higher levels of activity in the right olfactory bulb and left piriform cortex (but see Patzke et al., 2010).

The olfactory cortex, medial striatum, and hippocampal formation have been repeatedly shown to be involved in navigation (e.g., Papi and Casini, 1990; Shimizu et al., 2004; Gagliardo et al., 2005, 2007; Nardi and Bingman, 2007; Patzke et al., 2010; Jorge et al., 2014, 2016). Hippocampal involvement in homing pigeon navigation is thought reflect the use of landmarks and landscape features (Gagliardo et al., 1999). Surprisingly, however, no thalamic signal has been associated with homing. Specifically, despite abundant evidence in mammals that the anterior thalamic nuclei (Potegal, 1982; Taube, 1995; Stackman and Taube, 1997; Yoder et al., 2011; Clark and Taube, 2012; Jankowski et al., 2013) and lateral dorsal thalamic nuclei (Mizumori and Williams, 1993) play a central role in navigation, neither the anterior nor lateral dorsal thalamus, and by implication possible “head direction” cells (HDCs) in those regions have been reported to participate in avian navigation. HDCs found in the anterior and lateral dorsal thalamus of rodents (Mizumori and Williams, 1993; Taube, 1995) are thought to be part of a HDC system that integrates vestibular and visual input, underlying perception of movement through the environment (Potegal, 1982; Stackman and Taube, 1997; Yoder et al., 2011; Clark and Taube, 2012; Jankowski et al., 2013).

In the current study of homing pigeons, differential immediate early gene (IEG) expression was used to map patterns of neuronal activity associated with exposure to olfactory cues to investigate a possible role of the anterior thalamic nuclei in navigation. We compared the patterns of neuronal activity in young pigeons that were exposed either during displacement to the release site (“released” birds) or during simulated displacement (“home” birds) to natural or artificial odors, or to filtered air with no odors. Because the artificial odors used in this study did not provide the birds with navigational information (see Jorge et al., 2009), similar effects of natural and artificial odors on neuronal activity in the anterior thalamic nuclei are likely to result from olfactory activation of navigational circuits underlying homing (e.g., both types of odors should signaling “NOT at home, pay attention to cues of the movement”). Conversely, neuronal activity in the thalamic nuclei triggered by natural odors, but not artificial odors, suggests neuronal processing of navigational olfactory information (e.g., only natural odors can/should signaling information about spatial position). The findings reported here show, for the first time, that odor exposure in the context of homing differentially activates rostral dorsolateral neurons in the anterior thalamic nuclei and that these neurons seem to be particularly sensitive to naturally occurring odors encountered during the outward journey when path-based information about geographic position is used to determine the homeward direction.

## Methods

This study was carried out in accordance with the recommendations of the EU and the Portuguese law for animal welfare. All experimental protocols were approved by The Portuguese Veterinarian Commission under the reference PTDC/BIA-BEC/99416/2008.

### Subjects and procedures

Forty-eight young homing pigeons (7–8 weeks old) were given one of three olfactory treatments either during displacement to a remote site where they were subsequently released (released groups), or while remaining at the home loft (home groups).

“Released” and “home” pigeons were exposed to one of the following treatments in the transport containers: (1) pigeons were exposure to natural odors from the displacement or home loft (natural odor groups); (2) pigeons were exposure to a fixed sequence of artificial odors administered manually by opening valves on either sides of a small chamber containing a cotton swab to which an odor had been applied (100 μl of a single commercially available odor was used). Opening one pair of valves allowed filtered air to pass over one of the cotton swabs and carry the odor into the transportation box in which the pigeons were housed. After 5 min the valves controlling air flow to the first chamber were closed and the valves controlling air flow to the chamber containing a second odor were opened. In this way, odors were presented in 5 min intervals in the following order: lavender, camellia, eucalyptus, rose, and jasmine (artificial odor groups); and (3) pigeons were exposure to filtered air without odors. To produce the filtered air, natural environmental air was forced/drawn through charcoal filters that remove 99.9% of the odors present in the air (no-odor group). In an earlier study by Gagliardo et al. (2005), the pattern of neural activity in “released” and “home” birds exposed to “no odors” were indistinguishable, so in the present study only “released” birds were exposed to the no odor treatment. “Released” birds that returned home within 60–120 min, and “home” birds after a comparable period of time were euthanized and brains stained with c-Fos, IEG for immunohistochemical analysis of the brain tissue (*n* = 29).

Briefly, on each test day, 1–2 pigeons from each of the “released” treatments (see above) were displaced from the home loft (39°03′ N; 8°43′ W) to a familiar release site 9.8 km ESE of the loft, along with four additional individuals that received no treatment. Immediately after arriving at the release site, all pigeons in the “released” groups were placed in filtered air for 30 min. Eight minutes prior to release, the nostrils of all pigeons in the treatment groups were anesthetized with Xylocaine spray to prevent access to olfactory cues during the return flight. Then, all pigeons (treated and untreated) were released in a single flock. This procedure minimizes loss of pigeons and assures that most of the “released” pigeons arrive home in an appropriate time window to be perfused. In order to keep conditions as similar as possible between the “released” and the “home” treatment groups, “home” birds were placed in boxes similar to those used to transport the “released” birds, and they were exposed to a simulated outward journey; the transport boxes were wheeled around inside the loft to simulate the turns and associated disturbance experienced during the outward journey by the “released” birds for approximately 25 min. Then, the nostrils of “home” pigeons were anesthetized with Xylocaine. At the home loft both “home” and “released” pigeons were euthanized and perfused within 60–120 min after the start of the experiments (see also Jorge et al., 2009).

Pigeons were deeply anesthetized with an intra-peritoneal injection of sodium pentobarbital (0.5 ml per pigeon) and transcardially perfused with phosphate buffered saline (0.9% NaCl in 0.1 M phosphate buffer, pH 7.4) followed by fixative (4% paraformaldehyde in phosphate buffer—PB, pH 7.4). Brains were dissected and postfixed for an additional 24 h in the same fixative. For sectioning, the brains were first cryoprotected in sucrose buffer (30% sucrose in PB) and then embedded in sucrose–gelatin (30% sucrose, 10% gelatin in distilled water). The embedded brains were sectioned on a freezing microtome in the coronal plane at a thickness of 60 μm. Free-floating sections were stored in PB containing 0.001% of sodium azide at 4°C until they were stained. The immunohistochemical detection of c-Fos was performed with free-floating sections according to a previously published protocol in Jorge et al. (2014).

**Figure 1:**
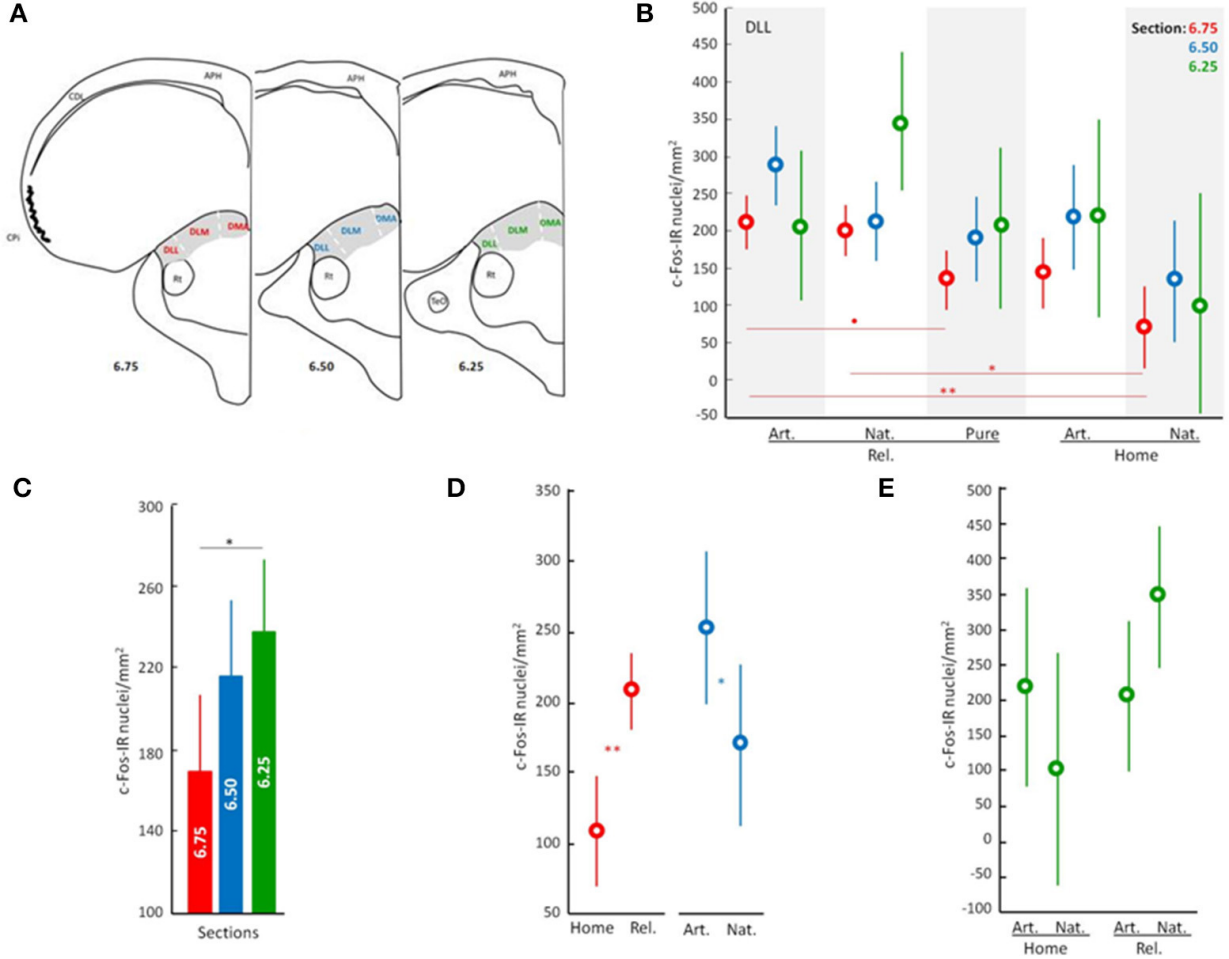
Overview of neuronal activity patterns in the anterior thalamic nucleus. Line drawing/Scheme **(A)** showing position of the anterior thalamic nucleus in gray and its subdivisions: DMA, dorsomedial anterior; DLM, dorsolateral anterior par medial; and DLL, dorsolateral anterior par lateral. APH, area parahippocampal; CDL, area corticoid dorsolateral; CPi, piriform cortex; Rt, nucleus rotundus; TeO, tectum optico Neuronal activity patterns of DLL according to the general effect of the treatment **(B)**, to the analyzed sections **(C)**, and to discriminated effects of the treatment **(D,E)**. Displacement effect includes birds displaced to the release site followed by the release (Rel.) and birds that stayed at home (Home). Odor effect includes birds supplied with artificial odors (Art.), natural odors (Nat.), or filtered air with no odors (Filtered). Error bars: Standard Deviations. Color discriminate sections: red, section at level A6.75; blue, section at level A6.50; and green, section at level A6.25. Significance is given by the *post-hoc* test Tukey's HLD for unequal sample sizes according to: ^•^*p* < 0.07, ^*^p < 0.05, ^**^p < 0.01, and ^***^p < 0.001.

C-Fos neuronal activation patterns occurring in the dorsal ATN following the different behavioral/olfactory treatments were analyzed. Three tissue sections from each of the three subdivisions of the dorsal ATN (dorsomedial anterior, DMA; dorsolateral anterior pars medialis, DLM; and dorsolateral anterior pars lateralis, DLL), were taken rostrally (atlas section A6.75 from Karten and Hoods (1967), medially (atlas section A6.50) and caudally (atlas section A6.25) and photographed at 8 × 40 magnification (Figure 1A, three sections for each of the three subdivisions). From these sections, c-Fos immunopositive nuclei were counted in a total of 162 representative counting frames (i.e., Nine counting frames for each section of each subdivision; frame area 0.0402 mm^2^). Photographs at equal light intensity were taken by a technician blind to experimental condition and converted to an 8-bit gray scale. c-Fos-immunopositive cells (cell nuclei with an optical density >150) were automatically counted using the ImageJ software (NIH, Bethesda, MD, USA).

### Statistical analyses

Labeled-neuron comparisons among groups were performed using a general linear model with repeated-measures analysis of variance.

Because there is no background information relative to the participation of the ATN nuclei in pigeon navigation, we initially performed two one-factor analyses to screen the data. One analysis was carried out to determine which subdivisions of the ATN, if any, were affected by the general treatment (“General treatment”: pigeons displaced and released exposed during the outward journey to natural air *n* = 8, artificial odors *n* = 7 or filtered air *n* = 7, and pigeons that stayed at home with natural air *n* = 4 or artificial odors *n* = 3). In this preliminary analysis, counts in distinct ATN subdivisions and levels were used as repeated measures (i.e., c-Fos immunopositive cell counts in DLL 6.25, DLM 6.25, DMA 6.25, DLL 6.50, DLM 6.50, DMA 6.50, DLL 6.75, DLM 6.75, and DMA 6.75). Significant interactions between treatment and the nuclei were only found for the DLL nucleus (see significance for DLL 6.75 in Table S1; but also, near significant results for DLL 6.50 and DLL 6.25). DLM and DMA did not show any significant interaction with the treatment (Table S1). The second analysis was carried out to determine whether there were differences in neuronal activation patterns in the selected area; based on the initial analysis, different anterior-posterior levels of DLL were targeted; see Figure 1B; “Brain slices”; DLL at slices A6.75 *n* = 28, A6.50 *n* = 28, and A6.25 *n* = 29. Significant differences were found between anterior and posterior levels of the DLL [*F*_(2, 82)_ = 3.47, *p* < 0.05; Figure 1C]. Thereafter, we focused the subsequent analyses on the neuronal activation patterns occurring at the three levels of the DLL subdivision of the ATN (6.75, 6.50 and 6.25).

First, we analyzed how “odors” (natural/artificial) and “displacement” (released/home) affected neuronal activation in the DLL using a two-factor analysis (“odors”; natural air, *n* = 11; artificial air, *n* = 11; and “Displacement,” released, *n* = 15; and home, *n* = 7). Section levels were treated as repeated measures. Then focusing on “released” birds, we analyzed how odor exposure, or its absence, affected neuronal activation in the DLL and examined possible hemispheric differences in neuronal activation using a two-factor analysis (“olfactory stimulus”: natural air, *n* = 8; artificial odors, *n* = 7; and filtered air, *n* = 7 and “hemisphere”: right, *n* = 22; and left, *n* = 22). Once again, section levels were treated as repeated measures. The *post-hoc* Tukey's HSD test was used for multiple comparisons with unequal sample sizes (Zar, 1999).

Neuronal activation patterns at the rostral, medial and caudal DLL levels were correlated with neuronal activation patterns at other brain nuclei including the right and left olfactory bulbs (BO), the right and left piriform cortex (CPi), the right and left area corticoidea dorsolateralis (CDL), the right and left hippocampal formation (broken down by three readily identifiable subdivisions: Nardi and Bingman, 2007; Atoji and Wild, 2014): TR, triangular; DM, dorsomedial; and DL, dorsolateral), the posterior dorsal thalamic nucleus (NDT, or its subdivisions: DMP, dorsomedial posterior; DIP, dorsointermedial posterior; and DLP, dorsolateral posterior); and the vestibular nuclei (NVe; including the subdivisions VeM, medial vestibular nucleus; and VeD, descending vestibular nucleus).

Labeled cell counts from all sampled brain regions were correlated with those recorded from the DLL to reveal any possible network interactions. A general linear model with multiple regression analysis was carried out to assess the statistical significance of the selected nuclei as predictors of the neuronal activation pattern observed in the DLL using a stepwise selection procedure (*P* ≤ 0.01 to add and *P* > 0.01 to remove). The final regression models complied with assumptions of multiple linear regression, all model residuals were normally distributed and multicollinearity of the predictors was examined by the tolerance and the Variance Inflation Factor (Zar, 1999).

All analyzes were carried out using STATISTICA 12 (Statsoft Inc., Tulsa, OK, USA).

## Results

Preliminary analyses pointed to the involvement of the dorsolateral lateral ATN (DLL) in navigation (see above; Figure 1B, Table S1), and suggested that different parts of the DLL may process different types of navigational information (see above; Figure 1C).

To better understand the contributions of different parts of the DLL, we analyzed the effects of the experimental treatment on patterns of neuronal activity (i.e., Displacement and Olfactory treatments). The analyses show that whether or not pigeons were displaced from the home loft (“released” vs. “home”) had a significant effect on neuronal activation patterns in the DLL (ANOVA; Interactions: Displacement, *p* < 0.001; Figure 1D, Table S2). There was also an interaction between displacement and olfactory exposure (ANOVA; Interactions: Displacement × Odors, *p* < 0.05; Figures 1D,E; Table S2). Moreover, the effect of displacement was particularly strong in the rostral DLL (section 6.75; ANOVA; Interactions: Displacement, *p* < 0.001; Figure 1D; Table S2), while an effect of odor exposure was more evident in the medial DLL (section A6.50; ANOVA; Interactions: Odors, *p* < 0.05; Figure 1D; Table S2). An interaction between treatments is suggested in the caudal DLL (section A6.25; ANOVA; Interactions: Displacement × Odors, *p* = 0.055; Figure 1E; Table S2).

**Figure 2:**
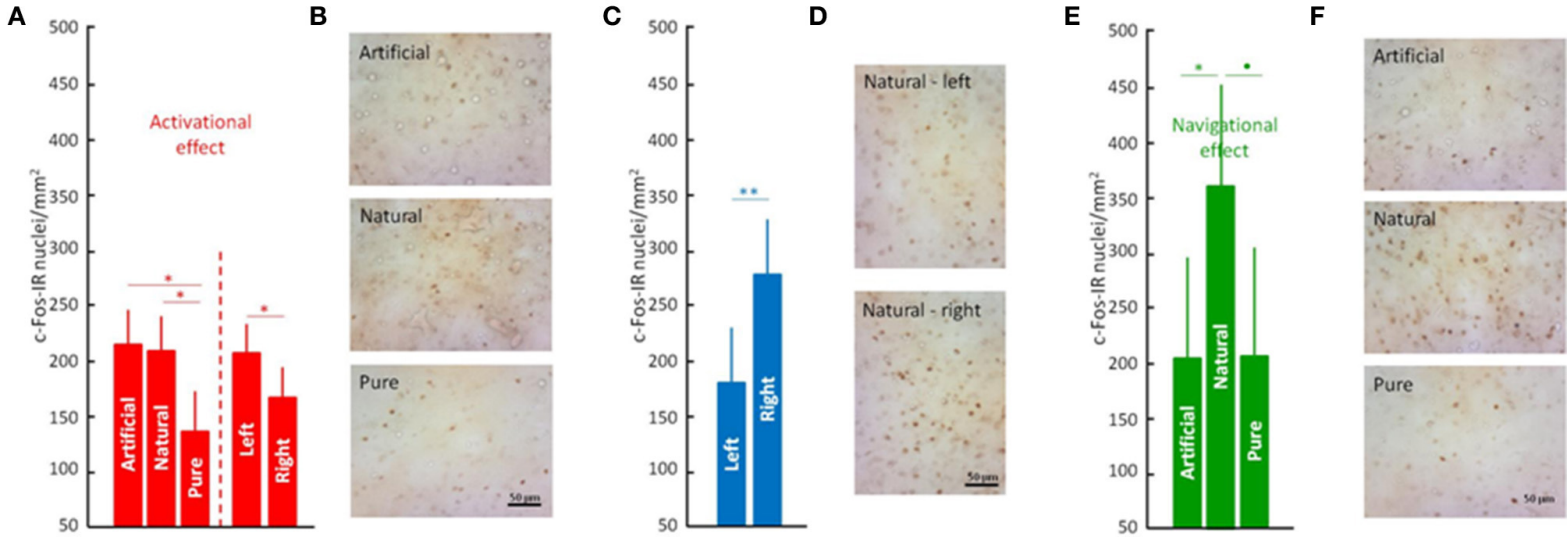
The anterior thalamus in avian navigation. Neuronal activity in the rostral **(A)**, medial **(C)**, and caudal **(E)** parts of the dorsolateral thalamus pars lateralis (DLL). Tissue samples showing c-Fos expression at rostral **(B)**, medial **(D)**, and caudal **(F)** DLL. Significance is given by the *post-hoc* test Tukey's HLD for unequal sample sizes according to: •*p* < 0.07, ^*^p < 0.05, and ^**^p < 0.01. Further legends as in Figure 1.

To address the question of whether or not odors are providing map/geographic position information, we reanalyzed the effects of access to odors (natural odors, artificial odors, or no odors) during the displacement to the release site (i.e., in birds that were released and homed). Furthermore, because odor involvement in navigation has been shown to be lateralized (Gagliardo et al., 2005, 2007; Patzke et al., 2010), we also considered a possible lateralization effect. Data confirmed the earlier findings showing that olfactory exposure affected neuronal activation patterns in the DLL (ANOVA; Interactions: Odors, *p* < 0.003 and Table S3) and also showed that the effects of odor exposure during the outward journey on neuronal activation are lateralized (ANOVA; Interactions: Lateralization, *p* < 0.0004; Figures 2A–D; Table S3). Specifically, the left rostral DLL displayed higher neuronal activation compared to the right, while the right medial DLL showed more intense neuronal activation than the left medial DLL. There was also an interesting contrast between the rostral and caudal DLL. In the rostral DLL, both groups with access to odors (i.e., natural and artificial) were significantly different from the group transported with no odors (Figures 2A,B), suggesting that neuronal activation in the rostral DLL was a general response to non-home odor exposure consistent with olfactory activation of the brain's navigational network (Jorge et al., 2014, 2016). In contrast, in the caudal DLL, exposure to natural odors, and the spatial/positional information they could convey (Gagliardo et al., 2005, 2007), produced the largest neuronal activational effect, implicating caudal DLL in the implementation of odor based, navigational mechanisms (Figures 2E,F).

**Figure 3:**
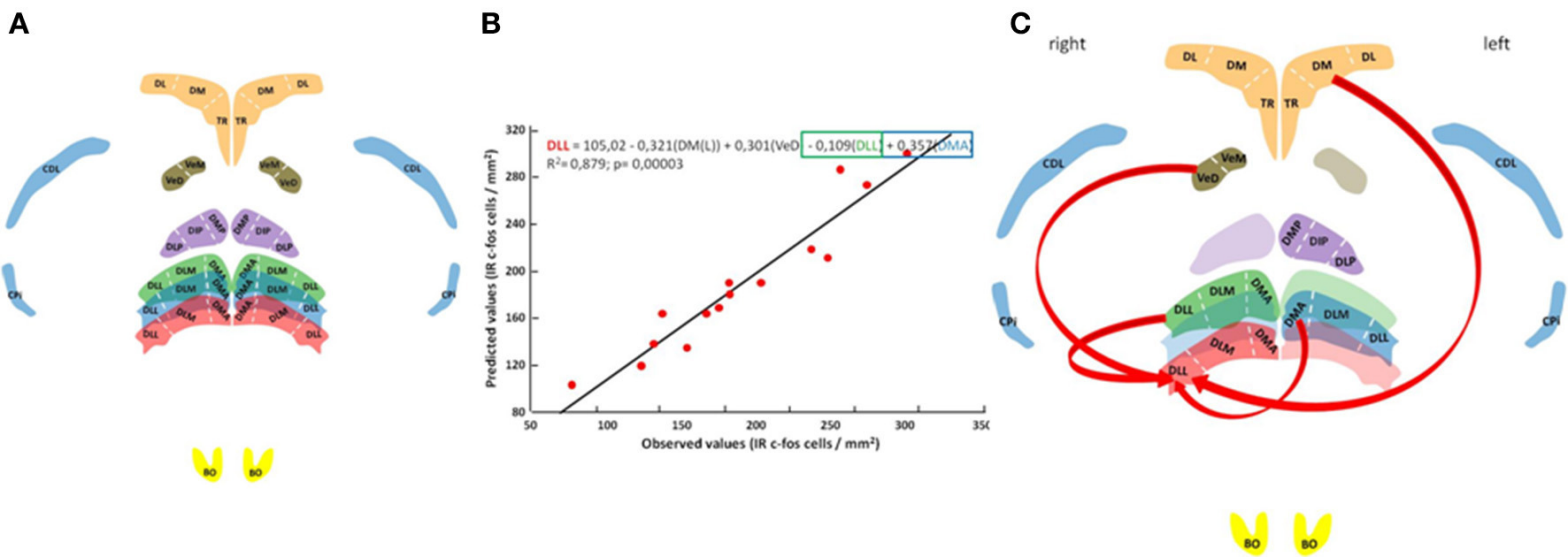
Neural pathway for thalamic navigation. Histology with anatomical reconstitution of brain nuclei analyzed **(A)**: Anterior thalamic nucleus subdivisions DMA, DLM, and DLL; posterior thalamic nucleus subdivisions DMP, DIP, and DLP; vestibular nucleus subdivisions VeM and VeD; parahippocampal subdivisions TR, DM, and DL; piriform cortex CPi; area corticoid dorsolateral CDL; and the olfactory bulbs BO. Color discriminate section: yellow, section A14.25; red, section A6.75; blue, section A6.50; green, section A6.25; orange, section A5.75; violet, section A5.25; and brown section P1.75. Observed vs. predicted values for the final model explaining neuronal activity in the rostral DLL **(B)**. The regression equation is presented on top followed by the *R*^2^ adjusted value and the overall significance given by the *F*-test. Schematic representation of rostral DLL predictors **(C)**.

In an attempt to place the observed neuronal activation patterns in the DLL (the rostral DLL in particular) in the broader context of the network of other brain areas thought to support homing pigeon navigation (Papi and Casini, 1990; Shimizu et al., 2004; Gagliardo et al., 2005, 2007; Nardi and Bingman, 2007; Patzke et al., 2010; Wu and Dickman, 2011; Jorge et al., 2014, 2016), neuronal activation patterns were subjected to correlational analysis (Figure 3). The results show that 88% of the total variation in labeled neurons occurring in the rostral DLL of released birds can be explained by the neuronal activation patterns occurring in the left dorsomedial region of the hippocampal formation, the descending vestibular nucleus, and some medial and caudal subdivisions of the ATN (Figures 3B,C; Table S4).

## Discussion

Understanding the range of functions of the dorsal thalamic nuclei in pigeons, inferred in part through differences in connectivity with the rest of the brain, is complicated by often subtle cytoarchitectural boundaries among the nuclei as well as regional differences within nuclei (Medina et al., 1997; Veenman et al., 1997). Nonetheless, the findings summarized in Figures 1–3 reveal a complex functional profile that reflects a specific role of the thalamic DLL in navigational processes supporting homing in pigeons.

The findings are consistent with two distinct roles of olfactory information in avian navigation. First, the non-specific activation of rostral DLL neurons caused by exposure to non-home odors (i.e., both natural and artificial; Figures 1D, 2A,B) could reflect odor-activated acquisition of non-olfactory, route-based information (e.g., vestibular, visual and/or proprioceptive; Jorge et al., 2009, 2010, 2014, 2016; Phillips and Jorge, 2014). Second, the olfactory activation of the medial DLL, which appears lateralized to the right hemisphere (Figures 2B,C) and is specific to naturally occurring odors that pigeons experienced along the displacement route, could reflect either use of familiar olfactory landmarks and/or of an olfactory map (Gagliardo et al., 2005; Wallraff, 2005, 2014) to determine the direction of home. The observation of lateralized activation in DLL is not surprising given that olfactory processing itself is lateralized (Gagliardo et al., 2005, 2007; Patzke et al., 2010). However, what is more difficult to explain is why different treatments resulted in more activation in the left rostral DLL on the one hand and the right medial DLL on the other (Figures 2A,C). Clearly more research is needed to be done to understand the origins of the differential, lateralized activation in DLL. However, that gap in understanding does not diminish the crucial findings of this study identifying for the first time an involvement of a thalamic region in avian navigation.

Evidence from previous studies of neuronal connectivity in pigeons indicate that the DLL comprises a major relay between the retina and the hyperpallium (Miceli et al., 2008) and is involved in processing visual information. More broadly, the dorsal thalamic nuclei receive afferent inputs from the dorsomedial hippocampal formation (Casini et al., 1986) and from the vestibular nuclei (Wu and Dickman, 2011). A connection with vestibular nuclei is consistent with a role of the thalamus in processing route-based, navigational information. In the context of an ATN connection with the vestibular system, it is tempting to speculate that the observed upregulation of neuronal activity in the DLL was a reflection of a head-direction signal used during the processing of route-based, outward journey information. Although thalamic head direction neurons are best known to reside in the anterior thalamic nuclei of rats (Taube, 1995), there are also HDCs in the rat lateral dorsal nucleus of the thalamus (Mizumori and Williams, 1993). Given the topographical similarity between the rat thalamic dorsal lateral nucleus and the pigeon DLL, the possibility that DLL provides a head direction signal along the lines described in rats is worth investigating. However, the presence of a hypothetical vestibular-head direction signal in DLL does not easily explain how DLL activity could modulate olfactory processing, or vice-versa. Nevertheless, the observed neuronal activity in the rostral DLL suggests that olfactory signals modulate rostral DLL neuronal activity (Figures 2A,B, 3B,C). Perhaps DLL neuronal activity could be involved in acquiring/processing outward journey information in support of navigation (e.g., generation of head direction signal; Winter et al., 2015a,b), which could then be coupled to the parallel processing of olfactory information.

However, to the best of our knowledge, there is no evidence that the olfactory bulbs or olfactory cortical regions project to DLL. Reciprocal connections have been reported linking the DMA with the piriform (olfactory) cortex (Bingman et al., 1994), as well as linking the DLM with much of the telencephalon including the ventral pallidum and motor processing striatal regions in both pigeons (Medina et al., 1997) and chicks (Csillag and Montagnese, 2005). In chicks, the DLM and DMA have been referred to as visceral and limbic thalamus, and of potential interest is a projection from DLM and DMA to reticular thalamic nuclei. Finally, a projection from the DLM to the hippocampus has also been reported in pigeons (Casini et al., 1986). It is worth noting then that the connectivity of the DLM (and DMA) to motor, limbic and hippocampal regions of the brain would suggest some role in influencing navigational processes, a role not detected in the observed pattern of c-Fos activation.

The open question that remains then is: what is it about the connectivity of the DLL that seemingly makes it relevant in supporting navigation in pigeons? The available data still suggest that the most salient aspect of pigeon DLL connectivity is that it resides in the middle of a major projection from the retina to the visual Wulst region of the hyperpallium as well as receiving inputs from the hyperpallial Wulst and optic tectum (Güntürkün et al., 1993; Miceli et al., 2008). In other words, the DLL, particularly the ventral portion, is intimately involved in processing visual information. Perhaps of more interest, in migratory Garden Warblers (*Sylvia borin*) the DLL has been implicated in a pathway that supports light-dependent compass orientation by the earth's magnetic field (Heyers et al., 2007). Given these findings, it is not surprising that neural activity in the DLL would be upregulated during navigation by homing pigeons.

Homing over short distances can be heavily reliant on the use of familiar, visual landmarks and the activation of the magnetic compass would also be expected. Of greater relevance here is why upregulated activity in the DLL observed in the present study should be modulated by olfactory exposure during transport to a release site. Many explanations are open, including the presence of familiar olfactory landmarks or sectors of an olfactory map previously geo-referenced with compass information relative to the home loft. Clearly further research is needed to understand how thalamic control of navigational processes, particularly those aspects that involve olfaction, is functionally integrated into the network of brain regions that support navigation.

## Author contributions

PJ and JP designed the research and analyzed the data. PJ and BP carried out field work. PJ, JP, and VB wrote the paper.

### Conflict of interest statement

The authors declare that the research was conducted in the absence of any commercial or financial relationships that could be construed as a potential conflict of interest.
