# Supplementary material for: Involvement of the Avian Dorsal Thalamic Nuclei in Homing Pigeon Navigation
Source: Front Behav Neurosci. 2017 Nov 2;11:213. doi: 10.3389/fnbeh.2017.00213 (PMC5674242; doi:10.3389/fnbeh.2017.00213)
Supplement: Supplementary file 1 [file Table1.DOCX]

**Table S1.** Overview of the general effect of the treatment on the neuronal activity of the ATN. Results from GLM analysis using a repeated-measure ANOVA. Main effect “Treatment” included 5 groups: Released/Artificial, Released/Natural, Released/Filtered, Home/Artificial, Home/Natural

| Effect | Test | Value | F | Effect | Error | P |
| --- | --- | --- | --- | --- | --- | --- |
| Intercept | Wilks | 0,014341 | 114,5536 | 9 | 15,00000 | 0,000000 |
| Treatment | Wilks | 0,016783 | 3,1814 | 36 | 57,94926 | 0,000043 |

UNIVARIATE RESULTS

| Effect | Degr. of freedom | SS  DLL (6.25) | MS  DLL (6.25) | F  DLL (6.25) | P  DLL (6.25) |
| --- | --- | --- | --- | --- | --- |
| Intercept | 1 | 1144444 | 1144444 | 74,39516 | 0,000000 |
| Treatment | 4 | 160531 | 40133 | 2,60885 | 0,062121 |
| Error | 23 | 353816 | 15383 |  |  |
| Total | 27 | 514347 |  |  |  |
|  |  | **DLM (6.25)** | **DLM (6.25)** | **DLM (6.25)** | **DLM (6.25)** |
| Intercept | 1 | 3240703 | 3240703 | 175,8921 | 0,000000 |
| Treatment | 4 | 82608 | 20652 | 1,1209 | 0,370952 |
| Error | 23 | 423761 | 18424 |  |  |
| Total | 27 | 506369 |  |  |  |
|  |  | **DMA (6.25)** | **DMA (6.25)** | **DMA (6.25)** | **DMA (6.25)** |
| Intercept | 1 | 2354348 | 2354348 | 210,0903 | 0,000000 |
| Treatment | 4 | 59678 | 14920 | 1,3313 | 0,288286 |
| Error | 23 | 257746 | 11206 |  |  |
| Total | 27 | 317424 |  |  |  |
|  |  | **DLL (6.50)** | **DLL (6.50)** | **DLL (6.50)** | **DLL (6.50)** |
| Intercept | 1 | 1050889 | 1050889 | 181,4780 | 0,000000 |
| Treatment | 4 | 60948 | 15237 | 2,6313 | 0,060512 |
| Error | 23 | 133187 | 5791 |  |  |
| Total | 27 | 194135 |  |  |  |
|  |  | **DLM (6.50)** | **DLM (6.50)** | **DLM (6.50)** | **DLM (6.50)** |
| Intercept | 1 | 2907285 | 2907285 | 158,2969 | 0,000000 |
| Treatment | 4 | 74063 | 18516 | 1,0081 | 0,423656 |
| Error | 23 | 422419 | 18366 |  |  |
| Total | 27 | 496481 |  |  |  |
|  |  | **DMA (6.50)** | **DMA (6.50)** | **DMA (6.50)** | **DMA (6.50)** |
| Intercept | 1 | 2189700 | 2189700 | 123,4231 | 0,000000 |
| Treatment | 4 | 93139 | 23285 | 1,3125 | 0,294930 |
| Error | 23 | 408052 | 17741 |  |  |
| Total | 27 | 501192 |  |  |  |
|  |  | **DLL (6.75)** | **DLL (6.75)** | **DLL (6.75)** | **DLL (6.75)** |
| Intercept | 1 | 585203,3 | 585203,3 | 266,5683 | 0,000000 |
| Treatment | 4 | 60245,6 | 15061,4 | 6,8607 | 0,000868 |
| Error | 23 | 50492,4 | 2195,3 |  |  |
| Total | 27 | 110738,0 |  |  |  |
|  |  | **DLM (6.75)** | **DLM (6.75)** | **DLM (6.75)** | **DLM (6.75)** |
| Intercept | 1 | 2734329 | 2734329 | 133,2638 | 0,000000 |
| Treatment | 4 | 16285 | 4071 | 0,1984 | 0,936642 |
| Error | 23 | 471918 | 20518 |  |  |
| Total | 27 | 488202 |  |  |  |
|  |  | **DMA (6.75)** | **DMA (6.75)** | **DMA (6.75)** | **DMA (6.75)** |
| Intercept | 1 | 1966891 | 1966891 | 155,2357 | 0,000000 |
| Treatment | 4 | 11625 | 2906 | 0,2294 | 0,919074 |
| Error | 23 | 291418 | 12670 |  |  |
| Total | 27 | 303043 |  |  |  |
