# Supplementary material for: Involvement of the Avian Dorsal Thalamic Nuclei in Homing Pigeon Navigation
Source: Front Behav Neurosci. 2017 Nov 2;11:213. doi: 10.3389/fnbeh.2017.00213 (PMC5674242; doi:10.3389/fnbeh.2017.00213)
Supplement: Supplementary file 2 [file Table2.DOCX]

**Table S2**. Overview of the general effect of the experimental treatment on the neuronal activity of the ATN. Results from GLM analysis using a repeated-measure ANOVA. Main effects: “Displacement” include Home and Fly; and Odors include Artificial and Natural

| Effect | Test | Value | F | Effect | Error | P |
| --- | --- | --- | --- | --- | --- | --- |
| Intercept | Wilks | 0,008314 | 132,5256 | 9 | 10 | 0,000000 |
| Displacement | Wilks | 0,095307 | 10,5471 | 9 | 10 | 0,000504 |
| Odors | Wilks | 0,423668 | 1,5115 | 9 | 10 | 0,264062 |
| Displ X Odors | Wilks | 0,213988 | 4,0813 | 9 | 10 | 0,019400 |

UNIVARIATE RESULTS

| Effect | Degr. of freedom | SS  DLL (6.25) | MS  DLL (6.25) | F  DLL (6.25) | P  DLL (6.25) |
| --- | --- | --- | --- | --- | --- |
| Intercept | 1 | 894955,0 | 894955,0 | 49,25492 | 0,000002 |
| Displacement | 1 | 61928,5 | 61928,5 | 3,40831 | 0,081380 |
| Odors | 1 | 575,9 | 575,9 | 0,03170 | 0,860682 |
| Displ X Odors | 1 | 76185,2 | 76185,2 | 4,19294 | 0,055471 |
| Error | 18 | 327057,5 | 18169,9 |  |  |
| Total | 21 | 480650,9 |  |  |  |
|  |  | **DLM (6.25)** | **DLM (6.25)** | **DLM (6.25)** | **DLM (6.25)** |
| Intercept | 1 | 2352837 | 2352837 | 135,1428 | 0,000000 |
| Displacement | 1 | 23804 | 23804 | 1,3673 | 0,257536 |
| Odors | 1 | 10424 | 10424 | 0,5988 | 0,449099 |
| Displ X Odors | 1 | 23181 | 23181 | 1,3315 | 0,263634 |
| Error | 18 | 313380 | 17410 |  |  |
| Total | 21 | 391429 |  |  |  |
|  |  | **DMA (6.25)** | **DMA (6.25)** | **DMA (6.25)** | **DMA (6.25)** |
| Intercept | 1 | 1676714 | 1676714 | 140,6777 | 0,000000 |
| Displacement | 1 | 7343 | 7343 | 0,6161 | 0,442705 |
| Odors | 1 | 61 | 61 | 0,0052 | 0,943528 |
| Displ X Odors | 1 | 39705 | 39705 | 3,3313 | 0,084614 |
| Error | 18 | 214539 | 11919 |  |  |
| Total | 21 | 265361 |  |  |  |
|  |  | **DLL (6.50)** | **DLL (6.50)** | **DLL (6.50)** | **DLL (6.50)** |
| Intercept | 1 | 838415,2 | 838415,2 | 128,0198 | 0,000000 |
| Displacement | 1 | 23061,8 | 23061,8 | 3,5214 | 0,076892 |
| Odors | 1 | 32375,7 | 32375,7 | 4,9435 | 0,039232 |
| Displ X Odors | 1 | 0,9 | 0,9 | 0,0001 | 0,990555 |
| Error | 18 | 117883,9 | 6549,1 |  |  |
| Total | 21 | 173224,5 |  |  |  |
|  |  | **DLM (6.50)** | **DLM (6.50)** | **DLM (6.50)** | **DLM (6.50)** |
| Intercept | 1 | 2139836 | 2139836 | 107,4787 | 0,000000 |
| Displacement | 1 | 20120 | 20120 | 1,0106 | 0,328089 |
| Odors | 1 | 25420 | 25420 | 1,2768 | 0,273332 |
| Displ X Odors | 1 | 52588 | 52588 | 2,6414 | 0,121495 |
| Error | 18 | 358369 | 19909 |  |  |
| Total | 21 | 431143 |  |  |  |
|  |  | **DMA (6.50)** | **DMA (6.50)** | **DMA (6.50)** | **DMA (6.50)** |
| Intercept | 1 | 1712840 | 1712840 | 81,27239 | 0,000000 |
| Displacement | 1 | 16388 | 16388 | 0,77757 | 0,389508 |
| Odors | 1 | 43466 | 43466 | 2,06242 | 0,168123 |
| Displ X Odors | 1 | 63320 | 63320 | 3,00447 | 0,100131 |
| Error | 18 | 379356 | 21075 |  |  |
| Total | 21 | 467540 |  |  |  |

**Table 1** (cont.)

| Effect | Degr. of freedom | SS  DLL (6.75) | MS  DLL (6.75) | F  DLL (6.75) | P  DLL (6.75) |
| --- | --- | --- | --- | --- | --- |
| Intercept | 1 | 475155,6 | 475155,6 | 194,5706 | 0,000000 |
| Displacement | 1 | 46320,6 | 46320,6 | 18,9677 | 0,000381 |
| Odors | 1 | 8341,9 | 8341,9 | 3,4159 | 0,081069 |
| Displ X Odors | 1 | 4810,6 | 4810,6 | 1,9699 | 0,177477 |
| Error | 18 | 43957,3 | 2442,1 |  |  |
| Total | 21 | 95618,5 |  |  |  |
|  |  | **DLM (6.75)** | **DLM (6.75)** | **DLM (6.75)** | **DLM (6.75)** |
| Intercept | 1 | 2105171 | 2105171 | 104,4879 | 0,000000 |
| Displacement | 1 | 2045 | 2045 | 0,1015 | 0,753727 |
| Odors | 1 | 5228 | 5228 | 0,2595 | 0,616646 |
| Displ X Odors | 1 | 11739 | 11739 | 0,5826 | 0,455165 |
| Error | 18 | 362655 | 20148 |  |  |
| Total | 21 | 378883 |  |  |  |
|  |  | **DMA (6.75)** | **DMA (6.75)** | **DMA (6.75)** | **DMA (6.75)** |
| Intercept | 1 | 1479837 | 1479837 | 138,6044 | 0,000000 |
| Displacement | 1 | 9097 | 9097 | 0,8521 | 0,368175 |
| Odors | 1 | 3402 | 3402 | 0,3186 | 0,579399 |
| Displ X Odors | 1 | 116 | 116 | 0,0109 | 0,917973 |
| Error | 18 | 192181 | 10677 |  |  |
| Total | 21 | 203738 |  |  |  |
