# Supplementary material for: Involvement of the Avian Dorsal Thalamic Nuclei in Homing Pigeon Navigation
Source: Front Behav Neurosci. 2017 Nov 2;11:213. doi: 10.3389/fnbeh.2017.00213 (PMC5674242; doi:10.3389/fnbeh.2017.00213)
Supplement: Supplementary file 3 [file Table3.DOCX]

**Table S3**. Overview of the general effect of “lateralization” and “odors” on the neuronal activity of the ATN. Results from GLM analysis using a repeated-measure ANOVA. Main effects: “Displacement” include Home and Fly; and “Lateralization” include Right and Left sides

| Effect | Test | Value | F | Effect | Error | P |
| --- | --- | --- | --- | --- | --- | --- |
| Intercept | Wilks | 0,074463 | 132,5819 | 3 | 32 | 0,000000 |
| Odors | Wilks | 0,544896 | 3,7835 | 6 | 64 | 0,002753 |
| Lateralization | Wilks | 0,567540 | 8,1279 | 3 | 32 | 0,000365 |
| Odors X Lateral | Wilks | 0,809220 | 1,1909 | 6 | 64 | 0,322555 |

UNIVARIATE RESULTS

| Effect | Degr. of freedom | SS  DLL (6.25) | MS  DLL (6.25) | F  DLL (6.25) | P  DLL (6.25) |
| --- | --- | --- | --- | --- | --- |
| Intercept | 1 | 2663405 | 2663405 | 96,34977 | 0,000000 |
| Odors | 2 | 223809 | 111904 | 4,04818 | 0,026484 |
| Lateralization | 1 | 22289 | 22289 | 0,80630 | 0,375532 |
| Odors X Lateral | 2 | 73113 | 36557 | 1,32245 | 0,279842 |
| Error | 34 | 939865 | 27643 |  |  |
| Total | 39 | 1258841 |  |  |  |
|  |  | **DLL (6.50)** | **DLL (6.50)** | **DLL (6.50)** | **DLL (6.50)** |
| Intercept | 1 | 2106492 | 2106492 | 188,5063 | 0,000000 |
| Odors | 2 | 69727 | 34864 | 3,1199 | 0,057016 |
| Lateralization | 1 | 94646 | 94646 | 8,4697 | 0,006329 |
| Odors X Lateral | 2 | 21708 | 10854 | 0,9713 | 0,388840 |
| Error | 34 | 379938 | 11175 |  |  |
| Total | 39 | 572766 |  |  |  |
|  |  | **DLL (6.75)** | **DLL (6.75)** | **DLL (6.75)** | **DLL (6.75)** |
| Intercept | 1 | 1386285 | 1386285 | 383,1336 | 0,000000 |
| Odors | 2 | 45135 | 22568 | 6,2371 | 0,004926 |
| Lateralization | 1 | 15304 | 15304 | 4,2296 | 0,047465 |
| Odors X Lateral | 2 | 2333 | 1166 | 0,3224 | 0,726613 |
| Error | 34 | 123022 | 3618 |  |  |
| Total | 39 | 186757 |  |  |  |
