# Supplementary material for: Involvement of the Avian Dorsal Thalamic Nuclei in Homing Pigeon Navigation
Source: Front Behav Neurosci. 2017 Nov 2;11:213. doi: 10.3389/fnbeh.2017.00213 (PMC5674242; doi:10.3389/fnbeh.2017.00213)
Supplement: Supplementary file 4 [file Table4.DOCX]

**Table S4**. Correlation analysis for DLL neuronal activation patterns occurring at section 6.75

| Depend. Variable | Test of SS Whole Model vs. SS Residual | | | | | | | | | | | | | | | | | | | | | |  |
| --- | --- | --- | --- | --- | --- | --- | --- | --- | --- | --- | --- | --- | --- | --- | --- | --- | --- | --- | --- | --- | --- | --- | --- |
|  | \| Multipl. R \| \| --- \| | | \| Multipl. R² \| \| --- \| | | \| Adjust. R² \| \| --- \| | | \| SS Model \| \| --- \| | | \| df Model \| \| --- \| | | \| MS Model \| \| --- \| | | \| SS Resid. \| \| --- \| | | \| df Resid. \| \| --- \| | | \| MS Resid. \| \| --- \| | | \| F \| \| --- \| | | \| P \| \| --- \| | |  |
| \| DLL (6.75) \| \| --- \| | 0,956 | | 0,914 | | 0,879 | | 49315,1 | | 4 | | 12328,77 | | 4650,466 | | 10 | | 465,047 | | 26,511 | | 0,00003 | |  |
| Effect | | Parameter Estimates Sigma-restricted parameterization | | | | | | | | | | | | | | | | | | | | | |
|  |  | \| Comment (B/Z/P) \| \| --- \| | | \| DLL (6.75) Param. \| \| --- \| | | \| DLL (6.75) Std.Err \| \| --- \| | | \| DLL (6.75) t \| \| --- \| | | \| DLL (6.75) p \| \| --- \| | | \| -95,00% Cnf.Lmt \| \| --- \| | | \| +95,00% Cnf.Lmt \| \| --- \| | | \| DLL (6.75) Beta (ß) \| \| --- \| | | \| DLL (6.75) St.Err.ß \| \| --- \| | | \| -95,00% Cnf.Lmt \| \| --- \| | | \| +95,00% Cnf.Lmt \| \| --- \| | |
| \| Interc. \| \| --- \| | |  | | 105,019 | | 26,840 | | 3,913 | | 0,003 | | 45,216 | | 164,823 | |  | |  | |  | |  | |
| \| DL_L \| \| --- \| | | Pooled | |  | |  | |  | |  | |  | |  | |  | |  | |  | |  | |
| \| DL_R \| \| --- \| | | Pooled | |  | |  | |  | |  | |  | |  | |  | |  | |  | |  | |
| \| BO_L \| \| --- \| | | Pooled | |  | |  | |  | |  | |  | |  | |  | |  | |  | |  | |
| \| BO_R \| \| --- \| | | Pooled | |  | |  | |  | |  | |  | |  | |  | |  | |  | |  | |
| \| Cpi_L \| \| --- \| | | Pooled | |  | |  | |  | |  | |  | |  | |  | |  | |  | |  | |
| \| Cpi_R \| \| --- \| | | Pooled | |  | |  | |  | |  | |  | |  | |  | |  | |  | |  | |
| \| TR_L \| \| --- \| | | Pooled | |  | |  | |  | |  | |  | |  | |  | |  | |  | |  | |
| \| TR_R \| \| --- \| | | Pooled | |  | |  | |  | |  | |  | |  | |  | |  | |  | |  | |
| \| DM_L \| \| --- \| | |  | | -0,321 | | 0,077 | | -4,186 | | 0,002 | | -0,492 | | -0,150 | | -0,417 | | 0,010 | | -0,639 | | -0,195 | |
| \| DM_R \| \| --- \| | | Pooled | |  | |  | |  | |  | |  | |  | |  | |  | |  | |  | |
| \| VeD \| \| --- \| | |  | | 0,301 | | 0,118 | | 2,556 | | 0,029 | | 0,039 | | 0,564 | | 0,241 | | 0,094 | | 0,031 | | 0,452 | |
| \| VeM \| \| --- \| | | Pooled | |  | |  | |  | |  | |  | |  | |  | |  | |  | |  | |
| \| DIP \| \| --- \| | | Pooled | |  | |  | |  | |  | |  | |  | |  | |  | |  | |  | |
| \| DLP \| \| --- \| | | Pooled | |  | |  | |  | |  | |  | |  | |  | |  | |  | |  | |
| \| DMP \| \| --- \| | | Pooled | |  | |  | |  | |  | |  | |  | |  | |  | |  | |  | |
| \| CDL_L \| \| --- \| | | Pooled | |  | |  | |  | |  | |  | |  | |  | |  | |  | |  | |
| \| CDL_R \| \| --- \| | | Pooled | |  | |  | |  | |  | |  | |  | |  | |  | |  | |  | |
| \| DLL (6.25) \| \| --- \| | |  | | -0,109 | | 0,048 | | -2,261 | | 0,047 | | -0,217 | | -0,002 | | -0,277 | | 0,123 | | -0,550 | | -0,004 | |
| \| DLM (6.25) \| \| --- \| | | Pooled | |  | |  | |  | |  | |  | |  | |  | |  | |  | |  | |
| \| DMA (6.25) \| \| --- \| | | Pooled | |  | |  | |  | |  | |  | |  | |  | |  | |  | |  | |
| \| DLM (6.50) \| \| --- \| | | Pooled | |  | |  | |  | |  | |  | |  | |  | |  | |  | |  | |
| \| DMA (6.50) \| \| --- \| | |  | | 0,357 | | 0,050 | | 7,109 | | 0,00003 | | 0,245 | | 0,469 | | 0,859 | | 0,121 | | 0,590 | | 1,128 | |
